# Placental pathology predicts infantile neurodevelopment

**DOI:** 10.1038/s41598-022-06300-w

**Published:** 2022-02-16

**Authors:** Megumi Ueda, Kenji J. Tsuchiya, Chizuko Yaguchi, Naomi Furuta-Isomura, Yoshimasa Horikoshi, Masako Matsumoto, Misako Suzuki, Tomoaki Oda, Kenta Kawai, Toshiya Itoh, Madoka Matsuya, Megumi Narumi, Yukiko Kohmura-Kobayashi, Naoaki Tamura, Toshiyuki Uchida, Hiroaki Itoh

**Affiliations:** 1 Department of Obstetrics and Gynecology, Hamamatsu University School of Medicine, Hamamatsu, Japan; 2 Research Center for Child Mental Development, Hamamatsu University School of Medicine, Hamamatsu, Japan

**Keywords:** Paediatric research, Intrauterine growth

## Abstract

The aim of present study was to investigate the association of placental pathological findings with infantile neurodevelopment during the early 40 months of life. 258 singleton infants were enrolled in the Hamamatsu Birth Cohort for Mothers and Children (HBC Study) whose placentas were saved in our pathological division. To assess the infantile neurodevelopment, we used Mullen Scales of Early Learning (gross motor, visual reception, fine motor, receptive language, expressive language) at 10, 14, 18, 24, 32, and 40 months. For obtaining placental blocks, we carried out random sampling and assessed eleven pathological findings using mixed modeling identified ‘Accelerated villous maturation’, ‘Maternal vascular malperfusion’, and ‘Delayed villous maturation’ as significant predictors of the relatively lower MSEL composite scores in the neurodevelopmental milestones by Mullen Scales of Early Learning. On the other hand, ‘Avascular villi’, ‘Thrombosis or Intramural fibrin deposition’, ‘Fetal vascular malperfusion’, and ‘Fetal inflammatory response’ were significant predictors of the relatively higher MSEL composite scores in the neurodevelopmental milestones by Mullen Scales of Early Learning. In conclusion, the present study is the first to report that some placental pathological findings are bidirectionally associated with the progression of infantile neurodevelopment during 10–40 months of age.

## Introduction

Epidemiological observations as well as experimental animal studies have collectively demonstrated the concept that environmental disruption during the early life stage affects health and diseases in later life^1–4^, based on which the Developmental Origin of Health and Diseases (DOHaD) theory was established^5^. Environmental homeostasis in utero is critical for the appropriate development of organs as well as homeostasis in entire biological systems after birth^1–4^.

The placenta is the largest organ in the pregnant uterus, connects the mother to the fetus, and supports most stages of fetal organogenesis via the transport of nutrients and gases and synthesis of hormones^6,7^. The placenta adapts to the maternal environment by changing its structure and function, thereby contributing to the maintenance of fetal development throughout the pregnant period^8^. The deterioration of placental transfer may result not only in an insufficient supply of nutrients and oxygen support, but also in various types of bioactive factors, such as hormones, immune substances, and protection against infection^9^. Increasing evidence has revealed that the condition of the placenta, including adaptations to the surrounding conditions, plays an important role in the in utero fetal programming process of health and risks of non-communicable diseases (NCDs) in offspring^6,10–13^. Khalief et al. reported that placental size negatively correlated with mental health in children and adolescents^14^. We recently demonstrated that the fetal/placental weight ratio was associated with the incidence of atopic dermatitis in female infants^15^. Barker et al. proposed the attractive concept of ‘Placental origins of chronic disease’^16^.

The placental pathology has historically been utilized in the assessment of placental conditions, including malfunction, and is, thus, referred to as the “memory of a pregnancy”^9^. The placental pathology reflects not only pathophysiological changes, but also physiological placental adaptations to various environmental factors from the maternal and fetal sides^9,17^. We recently showed that changes in the two-dimensional distribution profiles of specific lipids in the villi were responsible for pathologically abnormal placental findings using a two-dimensional imaging system based on matrix-assisted laser desorption/ionization-based mass spectrometry^18^. We subsequently reported that assisted reproductive technology affected the morphology of the placental basal plate^19^ and that placental thrombotic villous arterial lesions correlated with fetal cardiac functions measured by Doppler echocardiography^20^. Several large-scale studies examined the relationship between the placental histology and the outcomes of newborns in cases of severe intrauterine infections, preterm labor, and fetal hypoxia^21–24^; however, to the best of our knowledge, it currently remains unclear whether the placental pathology is a predictor of long-term outcomes in offspring, i.e. health and risks of diseases, in the general population.

**Figure 1:**
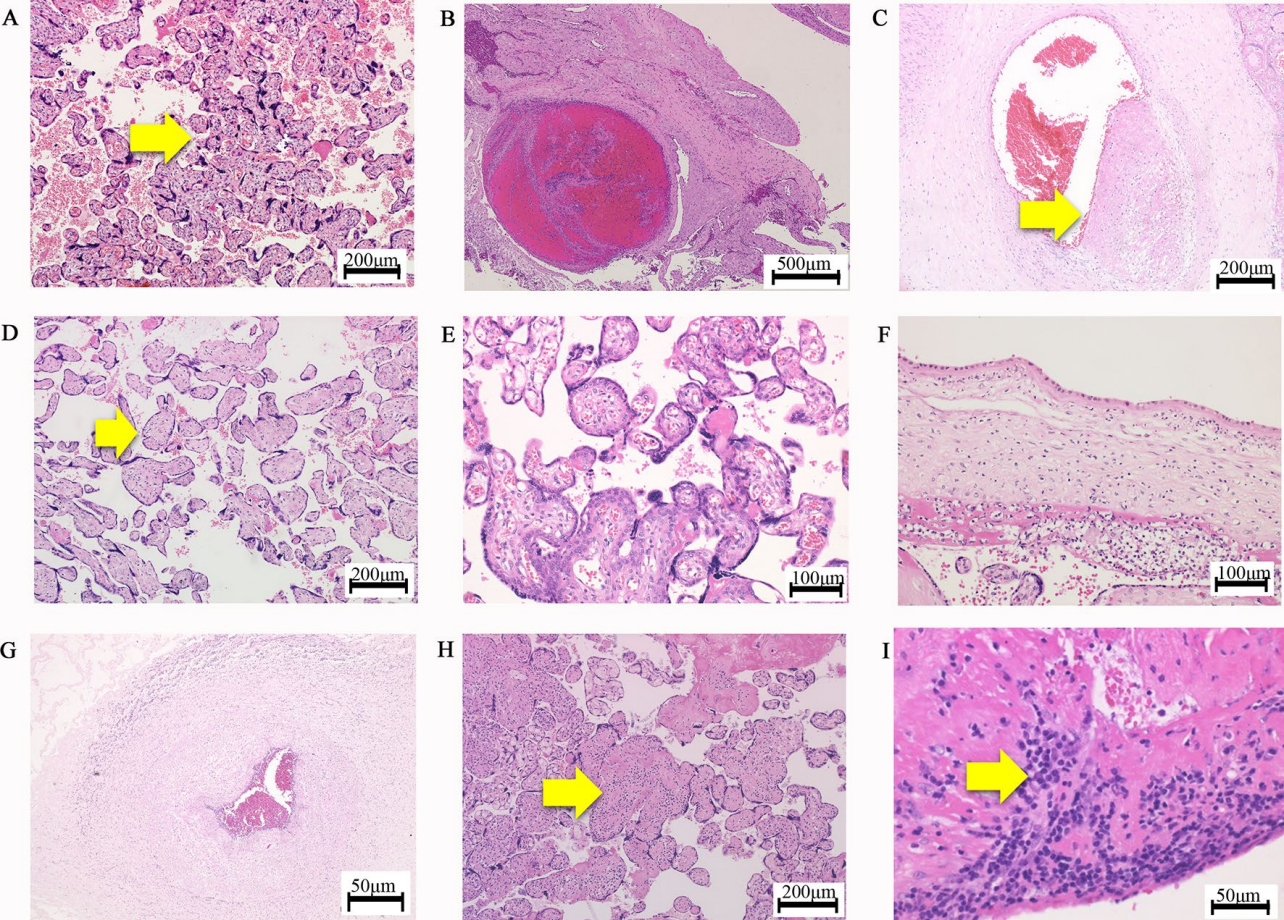
Representative pathological findings by HE staining of placentas. (**A**) ‘Accelerated villous maturation’; the yellow arrow indicates increases in the numbers of placental villi with the focal formation of tight adherent villous clusters with syncytial knots. (**B**) ‘Decidual arteriopathy’; A thrombus in decidual vessel. (**C**) ‘Thrombosis or Intramural fibrin deposition’; the yellow arrow indicates a fibrin cushion in the walls of stem villous vessels. (**D**) ‘Avascular villi’: the yellow arrow indicates a villus with hyalinized stroma, which is devoid of vessels. (**E**) ‘Delayed villous maturation’. (**F**) ‘Maternal inflammatory response’; the infiltration of neutrophils into the chorionic plate. (**G**) ‘Fetal inflammatory response’; the infiltration of neutrophils into the umbilical artery. (**H**) ‘VUE; villitis of unknown etiology’; the yellow arrow indicates lymphohistiocytic inflammation predominantly in the stroma of terminal villi. (**I**) ‘Deciduitis’; the yellow arrow indicates the infiltration of plasma cells.

Therefore, we hypothesized that the characteristics of the placental pathology are related to infantile physical and neuronal development in the Japanese population. The Hamamatsu Birth Cohort for Mothers and Children (HBC Study) was designed to elucidate the early developmental trajectories of children living in the community in Japan^25,26^. We recently performed a retrospective analysis of HBC study data and, among the placental pathological findings examined (Fig. 1, Tables 1, 2), identified ‘Maternal vascular malperfusion’ (Fig. 1A,B) as a significant predictor of a lower body weight and ‘Deciduitis’ (Fig. 1I) as a significant predictor of a small ponderal index, i.e. lean tendency, throughout the first 18 months of life using 258 whole placentas from singleton pregnancies, which were stored in our pathological division, among 1,258 pregnant women who were enrolled in the HBC study^27^. We planned the present study as a newly evolving investigation of the previous study and conducted a comprehensive analysis to identify the relationships between infantile neurodevelopment during 10–40 months of age, Mullen Scales of Early Learning (MSEL)^28^, and placental pathological findings using the same 258 whole placentas (Table1, 2) from singleton pregnancies in the HBC study.

**Table 1:** Pathological findings detected in 258 placentas.

Pathological findings	n	%
Accelerated villous maturation	67	26.0
Decidual arteriopathy	92	35.7
Thrombosis or Intramural fibrin deposition	76	29.5
Avascular villi	26	10.1
Delayed villous maturation	50	19.4
Maternal inflammatory response	103	39.9
Fetal inflammatory response	55	21.3
Villitis of unknown etiology	15	5.8
Deciduitis	14	5.4
Maternal vascular malperfusion	120	46.5
Fetal vascular malperfusion	86	33.3

**Table 2:** Perinatal backgrounds of parents and infants.

A							
	(Placenta not available) Not enrolled (n = 1000)			(Placenta available) Enrolled (n = 258)			*p*
	Mean or n	SD or %	Range	Mean or n	SD or %	Range	
**Parent characteristics**							
Maternal age (yr.)	31.1	4.96	(17–44)	32.9	5.24	(17–44)	< 0.001
Maternal body weight (non-pregnant) (kg)	51.9	8.68	(35.5–96)	53.7	11.29	(37.5–115)	0.007
Maternal BMI (non-pregnant) (kg/m^2^)	20.8	3.14	(14.6–37.6)	21.7	4.06	(15.9–40.4)	< 0.001
Body weight gain during pregnancy (kg)	10.7	4.35	(− 5.1–36.7)	9.9	4.5	(− 10.5–26.1)	0.01
Household income (million JPY/year)	6	2.84	(0.8–27)	6	2.8	(1–23)	0.79
Maternal education (Year)	13.8	1.97	(7–23)	13.8	2.06	(6–23)	0.59
Paternal age (yr.)	32.9	5.71	(18.9–62.6)	34.7	6.05	(18.9–52.7)	< 0.001
Hypertension during pregnancy	75	7.5%		28	10.9%		0.08
Diabetes mellitus	7	0.07%		7	2.7%		0.006
Postpartum depression (3 months after child birth)	124	12.4%		46	17.8%		0.023
**Infant characteristics**							
Birth weight (g)	2978.5	395.8	(946–4166)	2792.9	552.5	(1126–4286)	< 0.001
Gestational age at birth	39.1	1.48	(28–42)	38.4	1.93	(29–42)	< 0.001
Umbilical arterial pH	7.30	0.07	(6.77–7.49)	7.27	0.08	(6.83–7.49)	< 0.001
Singleton	962	96.2%		258	100%		0.001
Twin	38	3.8%		0	0%		

B							
		(Placenta not available) Not enrolled (N = 1000)		(Placenta available) Enrolled (N = 258)		*p*	
**Sex of newborns**							
Male		513		134		0.86	
Female		487		124			
**Parity**							
0		485		141		0.06	
1		390		77			
2+		125		40			
Term birth		949		223		0.001	
Preterm birth		51		35			
**Mode of delivery**							
Vaginal		824		87		< 0.001	
Vacuum Extraction		42		22			
Cesarean Section		126		149			

C							
		(Placenta available) Enrolled					
		Mean		SD		Range	
Placental weight (g)		530.9		119.66		(230–930)	
Placental area (cm^2^)		235.3		56.18		(230–930)	
Cord length (cm)		53.2		11.6		(27–90)	
Birth weight/Placental weight ratio (g/g)		5.357		0.877		(2.4–7.8)	

For hypertension during pregnancy, gestation diabetes mellitus, postpartum depression, singleton and twin, n and percentages (in parentheses) were described.

## Results

### Enrolled parturients and placentas

Table 1 summarizes the number of pathological findings detected in the placentas examined. Tables 2A,B summarizes the perinatal backgrounds of the participating mothers, fathers, and infants. Their association with infantile neurodevelopment was summarized in Supplemental Table 2 and Table 3. Table 2C summarizes placental measurements. Figure 1 shows the representative placental pathological findings examined.

**Table 3:** Mixed model analysis of Total Mullen Scales of Early Learning composite scores during 10 to 40 months in individual placental pathological findings.

**A: Individual pathological observations of the placenta**		
	Model 1	Model 2
	Coefficient (95% Conf. Interval)	Coefficient (95% Conf. Interval)
Accelerated villous maturation^a^	− 2.46 (− 4.30 to − 0.61)	− 2.57 (− 4.45 to − 0.69)
Decidual arteriopathy	− 1.24 (− 2.87 to 0.39)	− 0.37 (− 2.07 to 1.32)
Thrombosis or intramural fibrin deposition^b^	3.07 (1.36 to 4.79)	2.51 (0.72 to 4.31)
Avascular villi^b^	2.68 (0.15 to 5.21)	2.50 (− 0.13 to 5.13)
Delayed villous maturation^a^	− 2.62 (− 4.59 to − 0.64)	− 2.87 (− 4.83 to − 0.91)
Maternal inflammatory response	0.97 (− 0.69 to 2.63)	0.12 (− 1.74 to 1.98)
Fetal inflammatory response^b^	2.26 (0.25 to 4.28)	1.02 (− 1.31 to 3.36)
Villitis of unknown etiology	− 1.98 (− 5.07 to 1.12)	− 2.24 (− 5.41 to 0.93)
Deciduitis	− 0.46 (− 3.60 to 2.67)	− 0.89 (− 4.03 to 2.24)

**B: Conceptional pathological diagnosis of the placenta**		
	Model 1	Model 3
	Coefficient (95% Conf. Interval)	Coefficient (95% Conf. Interval)
Maternal vascular malperfusion^a^	− 2.09 (− 3.69 to − 0.50)	− 2.12 (− 3.71 to − 0.54)
Fetal vascular malperfusion^b^	3.41 (1.74 to 5.07)	3.43 (1.77 to 5.09)

Significance was set at a *p* value of 0.05 as described in the “Methods” section.

A; Adjusted for ‘Accelerated villous maturation’, ‘Decidual arteriopathy’, ‘Thrombosis or Intramural fibrin deposition’, ‘Avascular villi, ‘Delayed villous maturation’, ‘Maternal inflammatory response’, ‘Fetal inflammatory response’, ‘Villitis of unknown etiology (VUE)’, and ‘Deciduitis’. B; Adjusted for ’Maternal vascular malperfusion’ or ‘Fetal vascular malperfusion’. ’Maternal vascular malperfusion’ and ‘Fetal vascular malperfusion’ were separately assessed, because diagnostic criteria of them includes some of pathological findings listed in A.

The regression coefficients represent any changes associated with the presence/absence of the specific placental pathological changes, measured with the Mullen Scales of Early Learning composite score with the mean of 100 and the standard deviation of 15.

Model 1; Covariates included in the analysis were maternal parity, birth weight, and infantile sex, which may be related to placental pathological findings and MSEL composite scores.

Model 2; Additional covariates of all of other placental pathological findings.

Model 3; Additional covariate of all of Fetal vascular malperfusion or Maternal vascular malperfusion.

^a^Significant predictors of the relatively delayed achievement of neurodevelopmental milestones.

^b^Significant predictors of the relatively faster achievement of neurodevelopmental milestones.

### MSEL scores of five subscales

The MSEL composite scores were cellulated by the summation of net data of all five subscales, i.e. gross motor, visual reception, fine motor, receptive language, and expressive language^28^, at the ages of 10, 14, 18, 24, 32, and 40 months are described in Supplemental Tables.

### Placental pathology predictive of the relatively lower MSEL composite scores in the infantile neurodevelopmental milestones

**Figure 2:**
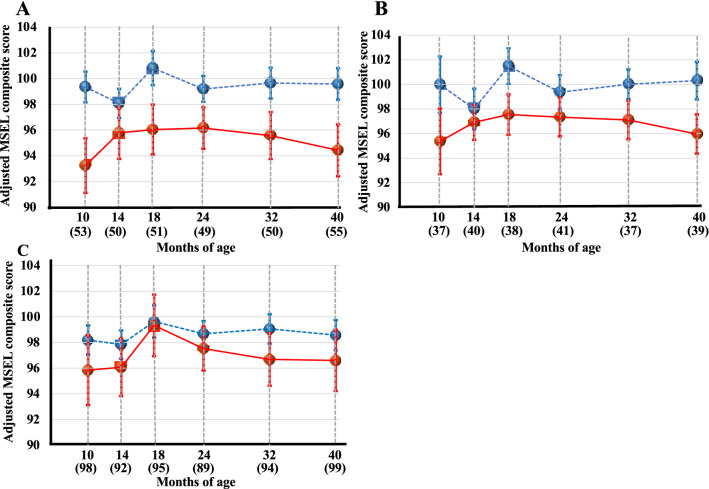
Chronological changes of Mullen Scales of Early Learning composite scores in each placental pathological finding during 10 to 40 months; ‘Accelerated villous maturation’ (**A**), ‘Delayed villous maturation’ (**B**), and ‘Maternal vascular malperfusion’ (**C**) as significant predictors of the relatively lower MSEL composite scores in the infantile neurodevelopmental milestones by a mixed model analysis. Red and blue dots indicate MSEL composite scores with and without ‘Accelerated villous maturation’, ‘Maternal vascular malperfusion’, or ‘Delayed villous maturation’, respectively. Circles and error bars indicate the mean and the standard error of the mean.

A mixed model analysis adjusted for potential confounders revealed that MSEL composite scores were significantly lower in infants with positive placental pathological findings of ‘Accelerated villous maturation’, ‘Maternal vascular malperfusion’, and ‘Delayed villous maturation’ than in those with negative placental pathological findings (Table 3, Fig. 2). Therefore, ‘Accelerated villous maturation’, ‘Delayed villous maturation’, and ‘Maternal vascular malperfusion’ were identified as significant predictors of the relatively lower MSEL composite scores in the infantile neurodevelopmental milestones during 10 to 40 months of age. There were no interactions between any of the above placental pathologies and neurodevelopmental outcomes with respect to age in months.

### Placental pathology predictive of the relatively higher MSEL composite scores in the infantile neurodevelopmental milestones

**Figure 3:**
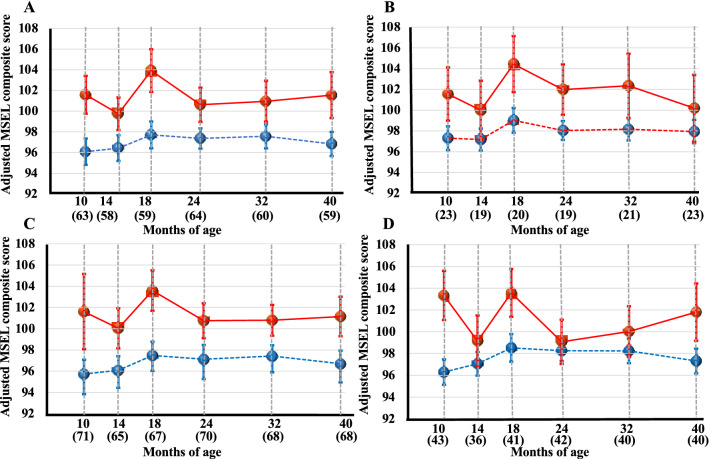
Chronological changes of Mullen Scales of Early Learning composite scores in each placental pathological finding during 10 to 40 months; ‘Thrombosis or Intramural fibrin deposition’ (**A**), ‘Avascular villi’ (**B**), ‘Fetal vascular malperfusion’ (**C**), and ‘Fetal inflammatory response’ (**D**) as significant predictors of the relatively higher MSEL composite scores in the infantile neurodevelopmental milestones by a mixed model analysis. Red and blue dots indicate MSEL composite scores with and without ‘Thrombosis or Intramural fibrin deposition’, ‘Avascular villi’, ‘Fetal inflammatory response’, and ‘Fetal vascular malperfusion’. Circles and error bars indicate the mean and standard error of the mean.

A mixed model analysis adjusted for potential confounders showed that MSEL composite scores were significantly higher in infants with positive placental pathological findings of ‘Thrombosis or Intramural fibrin deposition’, ‘Avascular villi’, ‘Fetal vascular malperfusion’, and Fetal inflammatory response’ than in those with negative placental pathological findings (Table 3, Fig. 3). Therefore, ‘Thrombosis or Intramural fibrin deposition’, ‘Avascular villi’, ‘Fetal inflammatory response’, and ‘Fetal vascular malperfusion’ were identified as significant predictors of the relatively higher MSEL composite scores in the infantile neurodevelopmental milestones during 10 to 40 months of age. There were no interactions between any of the above placental pathologies and neurodevelopmental outcomes with respect to age in months.

### Placental pathology with no relationship with infantile neurodevelopmental milestones

A mixed model analysis adjusted for potential confounders revealed that MSEL composite scores were similar in infants with positive placental pathological findings of ‘Decidual arteriopathy’, ‘Maternal inflammatory response’, ‘VUE’, and ‘Deciduitis’ and in those with negative placental pathological findings (Table 3).

## Discussion

The present results demonstrated that some pathological findings of the placenta were specifically connected to the achievement of the milestones of infantile neurodevelopment, in a bidirectional manner; i.e. relatively delayed and lower and higher MSEL composite scores (Table 3, Figs. 2, 3).

The pathological findings of ‘Accelerated villous maturation’, ‘Maternal vascular malperfusion’, and ‘Delayed villous maturation’ were significant predictors of the relatively lower MSEL composite scores in the infantile neurodevelopmental milestones during 10 to 40 months of age (Table 3, Fig. 2).

‘Accelerated villous maturation’ is an abnormal villous branching pattern that is diffusely observed in the placenta, and is suggested to be associated with hypoxic conditions in utero^18,29,30^. Previous studies indicated the strong suppressive effects of fetal hypoxia on physical development after birth in cases of severe fetal growth restrictions. The present study is the first to show that possible hypoxic conditions in utero represented by ‘Accelerated villous maturation’ are a predictor for the relatively lower scores of MSEL during 10 to 40 months of age (Table 3, Fig. 2A). ‘Accelerated villous maturation’ is a representative placental pathological finding in the recent concept of ‘Maternal vascular malperfusion’ by the Amsterdam Placental Workshop Group Consensus Statement^31^, which was also identified as a significant predictor of the relatively lower MSEL composite scores in the neurodevelopmental milestones during 10 to 40 months of age (Table 3, Fig. 2B).

We previously reported that the placental pathological finding of ‘Maternal vascular malperfusion’ was a predictor for a relatively light body weight during the first eighteen months of life^18^. Therefore, ‘Maternal vascular malperfusion’ predicts a relatively small body weight^18^ as well as the lower MSEL composite scores in the milestones of neurodevelopment during the infantile period. A potentially low supply of maternal blood into the placental intervillous space represented by the pathological finding of ‘Maternal vascular malperfusion’ may be causatively associated with suspected hypoxic conditions in fetal blood, which may contribute, at least partly, to the programming of a light body weight as well as relatively slow neurodevelopment during the early infantile period. The present results suggest a similar trajectory to that reported by Straugen et al., namely, the placental pathology of ‘Maternal vascular malperfusion’ was associated with autism spectrum disorder^32^. However, the pH of the umbilical arteries at delivery, representing acute changes during parturition, was not always associated with the detection of the chronic hypoxic findings of ‘Maternal vascular malperfusion’. Therefore, further physiological and epigenetic research is needed to prove this speculation.

The pathological finding of ‘Delayed villous maturation’ was also a predictor of the relatively lower MSEL composite scores in the infantile neurodevelopmental milestones during 10 to 40 months of age (Table 3, Fig. 2C). We cannot currently explain this relationship. However, the placental pathology of ‘Delayed villous maturation’ has frequently been reported in cases of maternal diabetic complications^9,17,31,33,34^; therefore, we speculate that chronic high glucose levels, clinical or subclinical, may have programmed the relatively lower MSEL composite scores in the neurodevelopmental milestones during the infantile period. A more detailed investigation is needed to prove this speculation.

In contrast, the pathological findings of ‘Thrombosis or Intramural fibrin deposition’, ‘Avascular villi’, ‘Fetal vascular malperfusion’, and ‘Fetal inflammatory response’ were significant predictors of the relatively higher MSEL composite scores in the infantile neurodevelopmental milestones during 10 to 40 months of age (Table 3, Fig. 3A–C).

The former two pathological findings of ‘Thrombosis or Intramural fibrin deposition’ and ‘Avascular villi’ indicate a localized and insufficient fetal blood supply to specific villus branches. ‘Thrombosis or Intramural fibrin deposition’ was observed inside the walls of large villus vessels and was regarded as an occlusion and/or the stenosis of upstream fetal blood flow in the specific villous area^9,17,31,34,35^. On the other hand, ‘Avascular villi’ was histologically defined as the total loss of villous capillaries and was regarded as an insufficiency of downstream fetal blood flow in the specific villous area^9,17,31,34,35^. Therefore, ‘Thrombosis or Intramural fibrin deposition’ and ‘Avascular villi’ both indicate the identical trajectory of a decreased fetal blood supply to specific and localized villi. ‘Thrombosis or Intramural fibrin deposition’ and ‘Avascular villi’ are representative placental pathological findings in the recent concept of ‘Fetal vascular malperfusion’ by the Amsterdam Placental Workshop Group Consensus Statement^31^.

Insufficient fetal blood supply to specific villous areas, i.e. ‘Thrombosis or Intramural fibrin deposition’, ‘Avascular villi’, and ‘Fetal vascular malperfusion’, were identified as significant predictors of the relatively higher MSEL composite scores in the infantile neurodevelopmental milestones during 10 to 40 months of age (Table 3, Fig. 3). The pathological finding of ‘Fetal inflammatory response’ was also a predictor of the relatively higher MSEL composite scores in the infantile neurodevelopmental milestones during 10 to 40 months of age (Table 3, Fig. 3D). It is paradoxical that pathological findings of an insufficient fetal blood supply to specific villous areas and fetal inflammatory response were corelated with relatively higher MSEL composite scores in the infantile neurodevelopmental milestones. We have no clear explanation and cannot fully deny the possible effect of unmeasured confounders. More detailed investigation is necessary.

The DOHaD concept highlights improvements in and/or interventions for perinatal, neonatal, and infantile care for the establishment of ‘preemptive medicine’ against the rapid spread of adult and senile NCDs^4,36^. It has been proposed as a central strategy of ‘preemptive medicine’ to identify high-risk individuals in early life as a promising target for earlier interventions^3,37,38^. Extensive efforts have been made over the past few decades to establish effective biomarkers through the application of ‘omics’ technologies, which may identify individuals at high risk of developing NCDs; however, only a very small number have been translated into routine health care support^39,40^. The present study revealed that some placental pathological findings were associated with changes in infantile neurodevelopment, in addition to our previous findings on body weight and body composition^27^ in the Japanese population, suggesting that placental pathological findings are applicable as a type of biomarker for predicting neuronal as well as physical development after birth. Follow-up investigations of the offspring of the present participants are now ongoing. However, more large-scale prospective cohort studies are needed.

There are some limitations in the present study. First of all, present sample size was small and we cannot deny the possibility that future large-scale study may lead to different associations from present ones, between placental pathology and progression of infantile neurodevelopment. We used the criteria of the Amsterdam Placental Workshop Group Consensus Statement, which includes subclassification of stage and/or grade^31^. However, we made assessment as positive vs negative for each individual pathological finding, without consideration of stage nor grade. The differential contribution of the stage and/or grade of each pathological finding is a future aim of the study. The diagnosis of ‘Delayed villous maturation’ and ‘Accelerated villous maturation’ are somewhat subjective even based on Amsterdam Placental Workshop Group Consensus Statement^31^. Placental pathological screening was carried out only pregnant women who agreed; resultantly, the number of cesarean deliveries was 149 among 258 subjects. Since majority of cesarean indication was elective cesarean section before onset of labor, average birth weight of 2815 g of the enrolled subject was somewhat smaller than Japanese average. The perinatal backgrounds of parents and infants of the rerolled subjects, who agreed to store the placentas, were not exactly identical to the unenrolled ones (Table 2). We enrolled only singleton pregnancies; while unenrolled group included twin progenies, which is one of the different backgrounds of two groups. Other difference in enrolled placentas were higher maternal age, higher maternal BMI, higher paternal age, higher incidence of Diabetes mellitus, higher incidence of postpartum maternal depression, lower birth weight, lower gestational age at birth, and lower umbilical arterial pH, than those in not enrolled ones, respectively (Table 2A). It is undeniable that such sampling bias (enrolled vs not enrolled) may induce Collider bias^41,42^, resulted in inflation or deflation of the magnitude of associations in some of the present analyzes.

In conclusion, the present study is the first to report that some placental pathological findings are bidirectionally associated with the progression of infantile neurodevelopment during 10–40 months of age.

## Methods

### Subjects

The present study was conducted as part of an ongoing cohort study (the HBC Study), which has been described elsewhere^25,26^. We consecutively contacted all pregnant women (n = 1,258) who were expected to give birth at our two research sites, the Hamamatsu University Hospital and Kato Maternity Clinic, which are both situated in Hamamatsu city, and who gave birth between 20 December 2007 and 31 October 2011. We previously confirmed that the enrolled parturients were representative of Japanese parturients in terms of age, socioeconomic status, parity, and the birthweight and gestational age of the child^25,26^. Among 1,258 subjects, we initially analyzed 261 whole placentas from singleton pregnancies because the parents had agreed to store their whole placentas in our pathological division. However, we excluded three placentas from the analysis: one infant had died, another had a confirmed diagnosis of Down’s syndrome with severe congenital heart disease, and the parents of the remaining infant refused to participate in this study after delivery. The remaining 258 (98.9%) placentas were analyzed.

All participating parturients were given a complete description of the present study, and provided written informed consent to participate. They were followed from entry into the study during mid-pregnancy to 40 months after childbirth.

### Preparation of placental tissue blocks

After weighing and an examination of gross morphology, whole placentas were stored in our pathological division after being vacuum-sealed in plastic packages with 10% formaldehyde (0.1 M sodium citrate buffer, pH 7.4). Seven paraffin blocks were systematically obtained from each placenta for the pathological examination by systematic random sampling, as previously described^18,19,27^. In brief, 5-mm-wide linear parallel slices of placental tissue were cut at an interval of approximately 3 cm perpendicular to the greatest dimension of the placental axis. All linear slices were vertically cut into small pieces at an interval of 3 cm. Seven blocks per placenta were obtained from seven randomly selected pieces of the placental parenchymal tissue obtained. Each block was made vertically from the maternal side to the fetal side. Two rolls of extraplacental membranes per placenta were together embedded in a block to make a single section. Each block was cut into 3-µm-thick sections and then subjected to hematoxylin and eosin (HE) staining. Eight sections (seven sections from the placental parenchyma and one from the extraplacental membrane) and two sections form umbilical cord were analyzed per placenta.

### Pathological examination

The pathological findings of placentas were classified into eleven categories with modifications from our recent study^27^, in consideration of the current Amsterdam Placental Workshop Group Consensus Statement^31^, i.e. ‘Accelerated villous maturation’; Fig. 1A, ‘Decidual arteriopathy’; Fig. 1B, ‘Thrombosis or Intramural fibrin deposition’; Fig. 1C, ‘Avascular villi’; Fig. 1D, ‘Delayed villous maturation; Fig. 1E, ‘Maternal inflammatory response’; Fig. 1F, ‘Fetal inflammatory response’; Fig. 1G, ‘Villitis of unknown etiology (VUE)’; Fig. 1H, ‘Deciduitis’; Fig. 1I.

(1) ‘Accelerated villous maturation’ was diagnosed as increased numbers of placental villi with the focal formation of tight adherent villous clusters^9,17,31,34^ typically with syncytial knots, increased perivillous fibrin, and the distal villous hypoplasia of small terminal villi^43^ (Fig. 1A), (2) ‘Decidual arteriopathy’ was diagnosed as vascular lesions including the fibrinoid necrosis of decidual vessels or arthrosis at the basal plate^9,31,34,44^ (Fig. 1B), (3) ‘Thrombosis or Intramural fibrin deposition’ was diagnosed as localized, protuberant mural lesions composed of proliferating fibroblasts intermixed with fibrin and erythrocytes in the walls of large placental vessels according to the description by Desa^9,17,31,34,35^ (Fig. 1C), (4) ‘Avascular villi’ was diagnosed as the total loss of villous capillaries and bland hyaline fibrosis in an older lesion^31^ (Fig. 1D), (5) ‘Delayed villous maturation’ was diagnosed as a monotonous villous population with reduced numbers of vasculosyncytial membranes, increases in the size of distal villi, increased numbers of stromal cells, and interstitial fluid uniformly distributed throughout the villous stroma^9,17,31,33,34^ (Fig. 1E), (6) ‘Maternal inflammatory response’ was diagnosed by the infiltration of neutrophils into the connective tissues of the chorionic plate and/or amnion basement membrane in the fetal surface of the placenta^9,17,31,34,45^ (Fig. 1F), (7) ‘Fetal inflammatory response’ was diagnosed by the infiltration of neutrophils into umbilical vessels or chorionic plate vessels^9,17,31,44^ (Fig. 1G), (8) ‘VUE’ was diagnosed by lymphohistiocytic inflammation predominantly localized to the villous stroma of terminal villi despite the absence of clinical symptoms of apparent infection in mothers or infants^9,17,31,34,46^ (Fig. 1H), and (9) ‘Deciduitis’ was employed as one of the findings of ‘others’, following the criteria of the Amsterdam Placental Workshop Group Consensus Statement^31^, which was diagnosed by the criteria of the presence of ≥ 50 lymphocytes/per high-power field^47^, often accompanied by chronic villitis or decidual necrosis^9,44^ (Fig. 1I). ‘Maternal vascular malperfusion’ and ‘Fetal vascular malperfusion’ were diagnosed inconsideration of the Amsterdam Placental Workshop Group Statement^31^. Maternal vascular a vascular malperfusion was diagnosed if ‘Accelerated villous maturation’ and/or ‘Decidual arteriopathy’ were observed. Fetal vascular malperfusion was diagnosed in case ‘Thrombosis or Intramural fibrin deposition’ and/or ‘Avascular villi’ were observed. In the present study, each of the eleven pathological findings were assessed as positive or negative through the majority decision of independent and blind surveys conducted by three researchers, i.e. Drs. Chizuko Yaguchi, Naomi Furuta, and Yoshimasa Horikoshi, as previously described^27^. They are researchers of placental pathology^18,27,48,49^.

### Assessment of infantile neurodevelopment

We used MSEL^28^ to evaluate neurodevelopment in infants. MSEL is a composite scale for assessing child development and comprises five subscales: gross motor, visual reception, fine motor, receptive language, and expressive language^28^. Measurements were performed when infants reached the ages of 10, 14, 18, 24, 32, and 40 months. Prior to follow-up assessments of the birth cohort, two experienced clinicians completed 3-month video training sessions, through which the agreement of their scoring of each item on the MSEL scale was attained. Separate 3-month video training sessions were set up and included an additional five assessors (child health professionals) who engaged in actual assessments. Developmental assessments with MSEL were conducted by referring to previously established data^28^. To evaluate the developmental trajectories of each of the five domains, MSEL-T scores, which are equivalent to Z-scores, but with a mean of 50 and standard deviation of 10, were generated.

Information on the demographic characteristics of mothers was collected during the pregnancy of enrolled parturients and included the age of the mother, parity, smoking, and pre-pregnancy height and weight^25,26^. Perinatal variables were collected from medical records^25,26^.

### Statistical analysis

MSEL composite scores^28^ at 10, 14, 18, 24, 32, and 40 months were set as dependent variables, and pathological findings as the independent variable. Continuous variables were reported as the mean ± SD. In comparisons of MSEL composite scores between two groups in the initial assessment (positive vs negative for each individual pathological finding), we performed the Student’s *t*-test or Mann–Whitney U test where appropriate. Significance was set at a *p* value of 0.05.

To assess the longitudinal trajectories of MSEL composite scores^28^, we adopted the method of mixed modeling^27^ using the *mixed* command provided by the generic statistical software, Stata version 16.1. Mixed modeling has the strength of analyzing longitudinal patterns of development in association with the fixed effect of the placental pathology that occurred long before anthropological measurements took place after birth^27^. Furthermore, mixed modeling allowed us to incorporate all available data into the analysis^27^ even if some data had missing values in the repeated measurements of MSEL composite scores. A growth curve model, i.e. mixed modeling with a random intercept and random slope^27^, was built for MSEL composite scores, during 10 to 40 months of age, adjusted for age in months at the time of infant assessments. We then incorporated all available covariates deemed to be potential confounders into the above analysis. Covariates included in the analysis were maternal parity, gestational weeks, and infantile sex, which may be related to placental pathological findings and MSEL composite scores^27^. Mixed model analyses of longitudinal data were conducted between 10 and 40 months in order to identify differential effects on MSEL composite scores between the presence and absence of several pathological findings. We subsequently entered all available indices of the placental pathology into the above analysis, shown as the final results. The marginal means and SD of MSEL composite scores, resulting from the significant effects of placental pathology findings, if confirmed, were calculated while averaging the effects of all covariates^27^. All *p* values were two-sided and significance was set at 0.05 for the mixed model analysis.

### Ethical considerations

The Ethics Committee of the Hamamatsu University School of Medicine approved all procedures (No. 20-82, 21-114, 22-29, 24-67, 24-237, 25-143, 25-283, E14-062, 17-037, and 20-233). All researches were performed in accordance with relevant guidelines and regulations which was designated by The Ethics Committee of the Hamamatsu University School of Medicine. Written informed consent was obtained from the participating parturients during pregnancy after a full explanation of the study.

## Supplementary Information

Supplementary Information.
