# Supplementary material for: Placental pathology predicts infantile neurodevelopment
Source: Sci Rep. 2022 Feb 16;12:2578. doi: 10.1038/s41598-022-06300-w (PMC8850429; doi:10.1038/s41598-022-06300-w)
Supplement: Supplementary file 1 — Supplementary Information. [file 41598_2022_6300_MOESM1_ESM.docx]

**Supplemental Table 1. Mean and SD of MSEL scores, stratified by negative/positive placental pathological findings of ‘Accelerated villous maturation’ (A), ‘Decidual arteriopathy’ (B), ‘Thrombosis or Intramural fibrin deposition’ (C), ‘Avascular villi’ (D), ‘Delayed villous maturation’ (E), ‘Maternal inflammatory response’ (F), ‘Fetal inflammatory response’ (G), ‘VUE’ (H), ‘Deciduitis’ (I), ‘Maternal vascular malperfusion’ (J), and ‘Fetal vascular malperfusion’ (K)**

**A**

| Placental pathological finding of ‘Accelerated villous maturation’ | | | | | | |
| --- | --- | --- | --- | --- | --- | --- |
| month | | Accelerated villous maturation | | Accelerated villous maturation | | P |
|  |  | negative | | positive | |  |
|  |  | mean | Std. Dev | mean | Std. Dev |  |
| MSEL composite score | 10 | 99.35 | 14.72 | 93.25 | 15.37 | 0.011 |
|  | 14 | 98.08 | 13.19 | 95.79 | 14.31 | 0.307 |
|  | 18 | 100.82 | 15.97 | 96.04 | 13.83 | 0.059 |
|  | 24 | 99.20 | 12.10 | 96.16 | 11.28 | 0.124 |
|  | 32 | 99.65 | 14.40 | 95.58 | 12.87 | 0.078 |
|  | 40 | 99.58 | 14.96 | 94.43 | 14.93 | 0.030 |

| Gross Motor  T-scores | 10 | 46.35 | 9.65 | 43.13 | 9.87 | 0.039 |
| --- | --- | --- | --- | --- | --- | --- |
|  | 14 | 48.57 | 11.17 | 45.42 | 11.61 | 0.088 |
|  | 18 | 47.87 | 9.719 | 49.15 | 10.71 | 0.420 |
|  | 24 | 48.37 | 9.29 | 44.93 | 8.52 | 0.021 |
|  | 32 | 48.31 | 10.36 | 46.97 | 11.32 | 0.447 |
|  | 40 | 48.87 | 10.93 | 46.48 | 11.47 | 0.171 |
| Fine Motor  T-scores | 10 | 48.78 | 9.72 | 45.38 | 10.36 | 0.032 |
|  | 14 | 47.52 | 10.30 | 46.61 | 10.98 | 0.589 |
|  | 18 | 49.28 | 10.29 | 50.21 | 9.25 | 0.571 |
|  | 24 | 48.50 | 10.32 | 45.68 | 9.30 | 0.078 |
|  | 32 | 48.96 | 10.80 | 46.76 | 10.48 | 0.212 |
|  | 40 | 50.41 | 11.16 | 46.90 | 11.11 | 0.046 |
| Visual Reception  T-scores | 10 | 48.51 | 10.76 | 45.77 | 11.39 | 0.117 |
|  | 14 | 48.83 | 9.42 | 48.34 | 8.54 | 0.739 |
|  | 18 | 48.99 | 9.85 | 45.28 | 8.48 | 0.016 |
|  | 24 | 49.67 | 10.62 | 46.73 | 9.26 | 0.073 |
|  | 32 | 48.74 | 10.02 | 46.05 | 9.44 | 0.097 |
|  | 40 | 50.53 | 10.56 | 46.89 | 11.04 | 0.031 |
| Expressive Language  T-scores | 10 | 48.46 | 9.37 | 46.51 | 8.91 | 0.188 |
|  | 14 | 49.14 | 10.25 | 48.09 | 10.62 | 0.533 |
|  | 18 | 48.98 | 10.02 | 46.58 | 11.00 | 0.147 |
|  | 24 | 49.55 | 10.51 | 45.96 | 12.05 | 0.044 |
|  | 32 | 49.20 | 9.78 | 46.25 | 10.70 | 0.074 |
|  | 40 | 49.48 | 10.95 | 47.29 | 10.93 | 0.201 |
| Receptive Language  T-scores | 10 | 48.51 | 9.33 | 45.19 | 10.22 | 0.031 |
|  | 14 | 48.78 | 9.20 | 47.88 | 9.37 | 0.555 |
|  | 18 | 48.83 | 10.00 | 45.28 | 8.57 | 0.023 |
|  | 24 | 49.45 | 10.67 | 48.40 | 10.78 | 0.550 |
|  | 32 | 50.12 | 9.44 | 47.37 | 9.13 | 0.078 |
|  | 40 | 49.51 | 11.21 | 46.51 | 10.13 | 0.080 |

**B**

| Placental pathological finding of ‘Decidual arteriopathy’ | | | | | | |
| --- | --- | --- | --- | --- | --- | --- |
| month | | Decidual arteriopathy | | Decidual arteriopathy | | P |
|  |  | negative | | positive | |  |
|  |  | mean | Std. Dev | mean | Std. Dev |  |
| MSEL composite score | 10 | 98.89 | 14.50 | 95.88 | 15.95 | 0.168 |
|  | 14 | 97.72 | 12.01 | 97.07 | 15.60 | 0.752 |
|  | 18 | 100.95 | 14.59 | 97.35 | 16.86 | 0.115 |
|  | 24 | 98.93 | 11.61 | 97.50 | 12.59 | 0.431 |
|  | 32 | 99.02 | 13.34 | 97.92 | 15.37 | 0.600 |
|  | 40 | 98.91 | 14.87 | 97.02 | 15.47 | 0.386 |
| Gross Motor  T-scores | 10 | 46.23 | 9.13 | 44.30 | 10.76 | 0.172 |
|  | 14 | 47.78 | 10.92 | 47.72 | 12.05 | 0.974 |
|  | 18 | 48.11 | 9.08 | 48.35 | 11.34 | 0.867 |
|  | 24 | 47.47 | 8.94 | 47.57 | 9.72 | 0.941 |
|  | 32 | 47.58 | 10.63 | 48.64 | 10.59 | 0.503 |
|  | 40 | 48.78 | 10.75 | 47.37 | 11.65 | 0.373 |
| Fine Motor  T-scores | 10 | 48.43 | 9.88 | 47.01 | 10.13 | 0.324 |
|  | 14 | 47.40 | 9.86 | 47.10 | 11.44 | 0.849 |
|  | 18 | 50.23 | 10.22 | 48.34 | 9.64 | 0.194 |
|  | 24 | 48.02 | 10.45 | 47.31 | 9.55 | 0.631 |
|  | 32 | 48.83 | 10.95 | 47.67 | 10.41 | 0.469 |
|  | 40 | 50.35 | 11.12 | 48.08 | 11.33 | 0.156 |
| Visual Reception  T-scores | 10 | 48.27 | 10.40 | 47.00 | 11.90 | 0.423 |
|  | 14 | 48.18 | 9.13 | 49.56 | 9.26 | 0.305 |
|  | 18 | 48.19 | 9.56 | 47.76 | 9.80 | 0.760 |
|  | 24 | 49.41 | 10.34 | 48.03 | 10.36 | 0.357 |
|  | 32 | 48.16 | 9.49 | 47.87 | 10.70 | 0.845 |
|  | 40 | 49.77 | 10.50 | 49.10 | 11.30 | 0.697 |
| Expressive Language  T-scores | 10 | 48.50 | 9.56 | 47.03 | 8.74 | 0.276 |
|  | 14 | 49.66 | 9.42 | 47.61 | 11.59 | 0.178 |
|  | 18 | 48.60 | 9.65 | 47.94 | 11.35 | 0.662 |
|  | 24 | 48.88 | 10.79 | 48.18 | 11.45 | 0.665 |
|  | 32 | 48.76 | 9.72 | 47.93 | 10.70 | 0.578 |
|  | 40 | 48.69 | 11.29 | 49.20 | 10.48 | 0.743 |
| Receptive Language T-scores | 10 | 48.10 | 9.52 | 46.90 | 9.90 | 0.393 |
|  | 14 | 48.62 | 8.99 | 48.40 | 9.65 | 0.872 |
|  | 18 | 48.59 | 9.37 | 46.75 | 10.28 | 0.197 |
|  | 24 | 50.05 | 10.18 | 47.62 | 11.44 | 0.127 |
|  | 32 | 49.26 | 8.47 | 49.70 | 10.89 | 0.752 |
|  | 40 | 49.03 | 10.82 | 48.19 | 11.31 | 0.592 |

**C**

| Placental pathological finding of ‘Thrombosis or intramural fibrin deposition’ | | | | | | |
| --- | --- | --- | --- | --- | --- | --- |
| month | | Thrombosis or  intramural fibrin deposition | | Thrombosis or  intramural fibrin deposition | | P |
|  |  | negative | | positive | |  |
|  |  | mean | Std. Dev | mean | Std. Dev. |  |
| MSEL composite score | 10 | 96.08 | 15.10 | 101.58 | 14.46 | 0.016 |
|  | 14 | 96.43 | 14.06 | 99.75 | 11.95 | 0.121 |
|  | 18 | 97.71 | 15.12 | 103.91 | 15.81 | 0.010 |
|  | 24 | 97.36 | 11.15 | 100.61 | 13.24 | 0.075 |
|  | 32 | 97.57 | 13.44 | 100.96 | 15.34 | 0.121 |
|  | 40 | 96.83 | 14.04 | 101.55 | 17.06 | 0.042 |
| Gross Motor  T-scores | 10 | 45.17 | 9.47 | 46.31 | 10.48 | 0.440 |
|  | 14 | 47.34 | 11.45 | 48.67 | 11.13 | 0.452 |
|  | 18 | 47.83 | 9.81 | 49.06 | 10.39 | 0.421 |
|  | 24 | 47.25 | 9.11 | 48.06 | 9.45 | 0.561 |
|  | 32 | 47.87 | 10.46 | 48.19 | 10.99 | 0.844 |
|  | 40 | 47.45 | 11.24 | 49.98 | 10.67 | 0.130 |
| Fine Motor  T-scores | 10 | 46.91 | 10.13 | 50.11 | 9.31 | 0.033 |
|  | 14 | 47.16 | 10.54 | 47.59 | 10.37 | 0.798 |
|  | 18 | 48.51 | 9.50 | 51.84 | 10.86 | 0.030 |
|  | 24 | 46.74 | 9.85 | 49.99 | 10.40 | 0.032 |
|  | 32 | 47.68 | 9.92 | 50.07 | 12.35 | 0.150 |
|  | 40 | 48.72 | 10.50 | 51.18 | 12.68 | 0.146 |
| Visual Reception  T-scores | 10 | 46.91 | 11.24 | 49.81 | 10.11 | 0.079 |
|  | 14 | 48.17 | 8.90 | 49.89 | 9.74 | 0.226 |
|  | 18 | 47.40 | 9.08 | 49.50 | 10.76 | 0.153 |
|  | 24 | 47.52 | 9.60 | 51.95 | 11.32 | 0.004 |
|  | 32 | 47.09 | 9.60 | 50.27 | 10.36 | 0.038 |
|  | 40 | 49.52 | 10.84 | 49.59 | 10.76 | 0.967 |
| Expressive Language  T-scores | 10 | 47.06 | 9.28 | 49.98 | 8.98 | 0.037 |
|  | 14 | 47.92 | 11.12 | 50.97 | 7.99 | 0.057 |
|  | 18 | 47.46 | 10.28 | 50.44 | 10.16 | 0.061 |
|  | 24 | 48.29 | 10.36 | 49.35 | 12.33 | 0.529 |
|  | 32 | 48.08 | 9.97 | 49.29 | 10.35 | 0.443 |
|  | 40 | 47.77 | 10.50 | 51.47 | 11.62 | 0.025 |
| Receptive Language  T-scores | 10 | 47.39 | 9.80 | 48.27 | 9.38 | 0.545 |
|  | 14 | 48.08 | 9.34 | 49.52 | 8.98 | 0.325 |
|  | 18 | 46.87 | 9.34 | 50.23 | 10.33 | 0.025 |
|  | 24 | 49.01 | 10.49 | 49.57 | 11.13 | 0.730 |
|  | 32 | 49.06 | 8.62 | 50.26 | 11.05 | 0.413 |
|  | 40 | 47.88 | 10.22 | 50.60 | 12.44 | 0.099 |

**D**

| Placental pathological finding of ‘Avascular villi’ | | | | | | |
| --- | --- | --- | --- | --- | --- | --- |
| month | | Avascular villi | | Avascular villi | | P |
|  |  | negative | | positive | |  |
|  |  | mean | Std. Dev | mean | Std. Dev |  |
| MSEL composite score | 10 | 97.30 | 15.37 | 101.55 | 12.30 | 0.203 |
|  | 14 | 97.18 | 13.62 | 99.99 | 12.40 | 0.390 |
|  | 18 | 99.02 | 15.83 | 104.44 | 12.08 | 0.140 |
|  | 24 | 98.05 | 12.05 | 101.98 | 10.59 | 0.174 |
|  | 32 | 98.16 | 14.07 | 102.35 | 14.15 | 0.199 |
|  | 40 | 97.94 | 15.07 | 100.19 | 15.38 | 0.501 |
| Gross Motor  T-scores | 10 | 45.60 | 9.81 | 44.94 | 9.72 | 0.762 |
|  | 14 | 47.80 | 11.48 | 47.41 | 10.32 | 0.885 |
|  | 18 | 47.95 | 10.03 | 50.38 | 9.41 | 0.291 |
|  | 24 | 47.39 | 9.067 | 48.49 | 10.52 | 0.605 |
|  | 32 | 48.43 | 10.38 | 44.37 | 11.81 | 0.091 |
|  | 40 | 48.40 | 10.92 | 46.87 | 12.64 | 0.533 |
| Fine Motor  T-scores | 10 | 47.57 | 10.06 | 50.62 | 9.00 | 0.167 |
|  | 14 | 47.17 | 10.52 | 48.30 | 10.14 | 0.648 |
|  | 18 | 48.91 | 9.66 | 54.78 | 11.73 | 0.011 |
|  | 24 | 47.55 | 10.05 | 49.57 | 10.70 | 0.378 |
|  | 32 | 48.01 | 10.29 | 51.57 | 13.70 | 0.143 |
|  | 40 | 49.29 | 11.12 | 50.88 | 12.23 | 0.524 |
| Visual Reception  T-scores | 10 | 47.55 | 10.90 | 49.89 | 11.49 | 0.336 |
|  | 14 | 48.73 | 9.32 | 48.42 | 8.02 | 0.887 |
|  | 18 | 47.56 | 9.68 | 52.05 | 8.38 | 0.043 |
|  | 24 | 48.36 | 10.24 | 53.54 | 10.32 | 0.026 |
|  | 32 | 47.66 | 10.12 | 51.24 | 7.57 | 0.110 |
|  | 40 | 49.19 | 10.78 | 52.38 | 10.65 | 0.182 |
| Expressive Language  T-scores | 10 | 47.56 | 9.18 | 51.10 | 9.54 | 0.084 |
|  | 14 | 48.41 | 10.57 | 52.85 | 6.88 | 0.068 |
|  | 18 | 48.26 | 10.47 | 49.17 | 8.94 | 0.709 |
|  | 24 | 48.45 | 11.19 | 50.41 | 9.16 | 0.462 |
|  | 32 | 48.29 | 10.16 | 49.80 | 9.41 | 0.517 |
|  | 40 | 48.86 | 10.89 | 49.16 | 11.74 | 0.901 |
| Receptive Language  T-scores | 10 | 47.64 | 9.70 | 47.82 | 9.45 | 0.932 |
|  | 14 | 48.37 | 9.28 | 49.89 | 8.93 | 0.489 |
|  | 18 | 47.76 | 9.87 | 48.94 | 8.75 | 0.601 |
|  | 24 | 48.84 | 10.91 | 52.27 | 7.87 | 0.175 |
|  | 32 | 49.41 | 9.40 | 49.49 | 9.75 | 0.971 |
|  | 40 | 48.61 | 11.10 | 49.47 | 10.28 | 0.727 |

**E**

| Placental pathological finding of ‘Delayed villous maturation’ | | | | | | |
| --- | --- | --- | --- | --- | --- | --- |
| month | | Delayed villous maturation | | Delayed villous maturation | | P |
|  |  | negative | | positive | |  |
|  |  | mean | Std. Dev | mean | Std. Dev |  |
| MSEL composite score | 10 | 98.20 | 14.75 | 95.85 | 16.60 | 0.394 |
|  | 14 | 97.85 | 13.33 | 96.07 | 14.18 | 0.463 |
|  | 18 | 99.63 | 15.78 | 99.33 | 14.76 | 0.915 |
|  | 24 | 98.68 | 12.22 | 97.53 | 10.97 | 0.587 |
|  | 32 | 99.06 | 14.46 | 96.68 | 12.46 | 0.356 |
|  | 40 | 98.57 | 15.16 | 96.61 | 14.87 | 0.466 |
| Gross Motor  T-scores | 10 | 45.90 | 9.82 | 43.85 | 9.56 | 0.246 |
|  | 14 | 47.61 | 11.30 | 48.34 | 11.61 | 0.716 |
|  | 18 | 48.25 | 9.99 | 47.98 | 10.06 | 0.884 |
|  | 24 | 47.37 | 8.77 | 48.03 | 10.82 | 0.683 |
|  | 32 | 47.71 | 10.70 | 49.07 | 10.25 | 0.491 |
|  | 40 | 47.79 | 11.25 | 50.15 | 10.36 | 0.232 |
| Fine Motor  T-scores | 10 | 48.08 | 9.75 | 47.13 | 11.02 | 0.597 |
|  | 14 | 47.30 | 10.32 | 47.22 | 11.11 | 0.964 |
|  | 18 | 49.66 | 10.39 | 48.88 | 8.36 | 0.664 |
|  | 24 | 47.66 | 10.19 | 48.16 | 9.91 | 0.776 |
|  | 32 | 48.17 | 10.85 | 49.41 | 10.33 | 0.529 |
|  | 40 | 49.51 | 11.25 | 49.27 | 11.29 | 0.903 |
| Visual Reception  T-scores | 10 | 47.93 | 10.74 | 47.28 | 12.02 | 0.744 |
|  | 14 | 48.86 | 8.98 | 48.10 | 9.98 | 0.635 |
|  | 18 | 47.99 | 9.73 | 48.16 | 9.33 | 0.922 |
|  | 24 | 48.63 | 10.65 | 49.99 | 9.07 | 0.448 |
|  | 32 | 48.02 | 10.15 | 48.20 | 8.98 | 0.921 |
|  | 40 | 49.92 | 10.52 | 47.93 | 11.87 | 0.302 |
| Expressive Language  T-scores | 10 | 48.49 | 9.21 | 45.54 | 9.27 | 0.080 |
|  | 14 | 49.52 | 9.94 | 46.33 | 11.52 | 0.082 |
|  | 18 | 48.60 | 10.30 | 47.25 | 10.40 | 0.466 |
|  | 24 | 48.93 | 10.76 | 47.46 | 11.98 | 0.445 |
|  | 32 | 49.07 | 10.20 | 45.79 | 9.15 | 0.075 |
|  | 40 | 49.22 | 10.96 | 47.48 | 10.97 | 0.372 |
| Receptive Language  T-scores | 10 | 47.70 | 9.52 | 47.47 | 10.37 | 0.896 |
|  | 14 | 48.52 | 9.11 | 48.59 | 9.79 | 0.967 |
|  | 18 | 47.79 | 9.72 | 48.25 | 9.99 | 0.800 |
|  | 24 | 49.22 | 10.96 | 49.09 | 9.70 | 0.945 |
|  | 32 | 49.73 | 9.69 | 48.11 | 8.15 | 0.346 |
|  | 40 | 48.93 | 11.11 | 47.73 | 10.55 | 0.541 |

**F**

| Placental pathological finding of ‘Maternal inflammatory response’ | | | | | | |
| --- | --- | --- | --- | --- | --- | --- |
| month | | Maternal inflammatory response | | Maternal inflammatory response | | P |
|  |  | negative | | positive | |  |
|  |  | mean | Std. Dev | mean | Std. Dev |  |
| MSEL composite score | 10 | 95.50 | 14.86 | 101.18 | 14.88 | 0.008 |
|  | 14 | 96.32 | 12.98 | 99.19 | 14.16 | 0.157 |
|  | 18 | 97.54 | 15.82 | 102.65 | 14.71 | 0.024 |
|  | 24 | 98.53 | 11.65 | 98.29 | 12.46 | 0.889 |
|  | 32 | 98.37 | 13.97 | 98.97 | 14.39 | 0.772 |
|  | 40 | 98.16 | 14.68 | 98.25 | 15.79 | 0.968 |
| Gross Motor  T-scores | 10 | 44.04 | 9.24 | 47.73 | 10.19 | 0.008 |
|  | 14 | 46.33 | 11.77 | 49.86 | 10.37 | 0.031 |
|  | 18 | 46.93 | 9.93 | 50.14 | 9.79 | 0.024 |
|  | 24 | 48.31 | 9.09 | 46.31 | 9.31 | 0.129 |
|  | 32 | 48.73 | 9.66 | 46.88 | 11.80 | 0.235 |
|  | 40 | 47.81 | 11.29 | 48.89 | 10.84 | 0.497 |
| Fine Motor  T-scores | 10 | 47.00 | 9.79 | 49.28 | 10.15 | 0.120 |
|  | 14 | 46.80 | 10.36 | 48.03 | 10.64 | 0.418 |
|  | 18 | 48.48 | 10.34 | 51.09 | 9.37 | 0.070 |
|  | 24 | 47.72 | 9.50 | 47.84 | 11.02 | 0.934 |
|  | 32 | 48.34 | 9.83 | 48.50 | 12.01 | 0.916 |
|  | 40 | 49.18 | 10.87 | 49.90 | 11.81 | 0.655 |
| Visual Reception  T-scores | 10 | 46.10 | 11.08 | 50.36 | 10.33 | 0.006 |
|  | 14 | 47.66 | 8.64 | 50.27 | 9.78 | 0.050 |
|  | 18 | 46.18 | 8.65 | 50.83 | 10.40 | 0.0007 |
|  | 24 | 49.28 | 9.31 | 48.36 | 11.74 | 0.531 |
|  | 32 | 47.56 | 9.27 | 48.78 | 10.82 | 0.396 |
|  | 40 | 49.59 | 10.34 | 49.48 | 11.51 | 0.945 |
| Expressive Language  T-scores | 10 | 46.46 | 8.64 | 50.19 | 9.78 | 0.005 |
|  | 14 | 48.64 | 10.64 | 49.22 | 9.89 | 0.706 |
|  | 18 | 47.67 | 10.78 | 49.35 | 9.54 | 0.258 |
|  | 24 | 48.75 | 10.99 | 48.45 | 11.09 | 0.853 |
|  | 32 | 48.89 | 9.97 | 47.82 | 10.25 | 0.468 |
|  | 40 | 48.96 | 10.86 | 48.78 | 11.17 | 0.909 |
| Receptive Language  T-scores | 10 | 47.30 | 9.67 | 48.20 | 9.66 | 0.515 |
|  | 14 | 48.16 | 8.89 | 49.10 | 9.75 | 0.494 |
|  | 18 | 47.04 | 9.72 | 49.17 | 9.71 | 0.131 |
|  | 24 | 49.70 | 10.11 | 48.43 | 11.51 | 0.414 |
|  | 32 | 49.70 | 9.15 | 49.01 | 9.84 | 0.620 |
|  | 40 | 48.78 | 10.13 | 48.61 | 12.25 | 0.914 |

**G**

| Placental pathological finding of ‘Fetal inflammatory response’ | | | | | | |
| --- | --- | --- | --- | --- | --- | --- |
| month | | Fetal inflammatory response | | Fetal inflammatory response | | P |
|  |  | negative | | positive | |  |
|  |  | mean | Std. Dev | mean | Std. Dev |  |
| MSEL composite score | 10 | 96.30 | 14.86 | 103.33 | 14.82 | 0.006 |
|  | 14 | 97.06 | 13.35 | 99.14 | 14.17 | 0.409 |
|  | 18 | 98.52 | 15.81 | 103.57 | 14.02 | 0.064 |
|  | 24 | 98.26 | 11.62 | 99.08 | 13.18 | 0.694 |
|  | 32 | 98.25 | 13.96 | 100.02 | 14.72 | 0.479 |
|  | 40 | 97.31 | 14.61 | 101.80 | 16.63 | 0.092 |
| Gross Motor  T-scores | 10 | 45.15 | 9.82 | 46.91 | 9.62 | 0.292 |
|  | 14 | 47.35 | 11.58 | 49.35 | 10.33 | 0.321 |
|  | 18 | 48.15 | 9.87 | 48.37 | 10.50 | 0.901 |
|  | 24 | 47.69 | 9.39 | 46.83 | 8.55 | 0.586 |
|  | 32 | 48.58 | 10.03 | 45.64 | 12.40 | 0.119 |
|  | 40 | 48.26 | 11.05 | 48.14 | 11.44 | 0.952 |
| Fine Motor  T-scores | 10 | 47.19 | 10.00 | 50.63 | 9.50 | 0.044 |
|  | 14 | 47.46 | 10.56 | 46.60 | 10.18 | 0.646 |
|  | 18 | 49.33 | 10.05 | 50.23 | 10.00 | 0.606 |
|  | 24 | 47.58 | 10.06 | 48.45 | 10.40 | 0.612 |
|  | 32 | 48.08 | 10.13 | 49.63 | 12.85 | 0.412 |
|  | 40 | 49.09 | 10.85 | 50.98 | 12.69 | 0.336 |
| Visual Reception  T-scores | 10 | 46.70 | 10.90 | 51.93 | 10.31 | 0.005 |
|  | 14 | 48.72 | 8.71 | 48.65 | 10.98 | 0.965 |
|  | 18 | 47.33 | 9.35 | 50.72 | 10.32 | 0.042 |
|  | 24 | 48.69 | 9.76 | 49.71 | 12.39 | 0.562 |
|  | 32 | 47.49 | 9.63 | 50.23 | 10.82 | 0.116 |
|  | 40 | 49.58 | 10.73 | 49.38 | 11.16 | 0.914 |
| Expressive Language  T-scores | 10 | 47.14 | 9.06 | 51.02 | 9.52 | 0.014 |
|  | 14 | 48.84 | 10.46 | 48.97 | 9.93 | 0.944 |
|  | 18 | 47.99 | 10.60 | 49.69 | 9.11 | 0.344 |
|  | 24 | 48.61 | 10.90 | 48.70 | 11.52 | 0.965 |
|  | 32 | 48.31 | 10.15 | 48.99 | 9.90 | 0.700 |
|  | 40 | 48.15 | 10.89 | 51.89 | 10.85 | 0.050 |
| Receptive Language  T-scores | 10 | 47.32 | 9.39 | 48.96 | 10.60 | 0.323 |
|  | 14 | 48.12 | 9.36 | 50.24 | 8.58 | 0.212 |
|  | 18 | 47.27 | 9.827 | 50.25 | 9.16 | 0.081 |
|  | 24 | 49.16 | 10.29 | 49.32 | 12.14 | 0.930 |
|  | 32 | 49.53 | 9.24 | 49.02 | 10.19 | 0.764 |
|  | 40 | 48.44 | 10.71 | 49.79 | 12.13 | 0.477 |

**H**

| Placental pathological finding of ‘VUE’ | | | | | | |
| --- | --- | --- | --- | --- | --- | --- |
| month | | VUE | | VUE | | P |
|  |  | negative | | positive | |  |
|  |  | mean | Std. Dev | mean | Std. Dev |  |
| MSEL composite score | 10 | 97.67 | 15.17 | 98.81 | 14.48 | 0.791 |
|  | 14 | 97.39 | 13.18 | 98.34 | 17.48 | 0.802 |
|  | 18 | 100.03 | 15.00 | 93.63 | 21.28 | 0.138 |
|  | 24 | 98.49 | 11.94 | 97.61 | 12.41 | 0.797 |
|  | 32 | 98.78 | 13.79 | 96.05 | 18.75 | 0.518 |
|  | 40 | 98.29 | 14.77 | 96.86 | 19.86 | 0.743 |
| Gross Motor  T-scores | 10 | 45.59 | 9.76 | 44.60 | 10.32 | 0.717 |
|  | 14 | 47.73 | 11.17 | 48.10 | 13.83 | 0.907 |
|  | 18 | 48.52 | 9.68 | 43.77 | 12.98 | 0.085 |
|  | 24 | 47.56 | 9.19 | 46.74 | 9.70 | 0.757 |
|  | 32 | 47.95 | 10.69 | 48.21 | 9.49 | 0.935 |
|  | 40 | 48.53 | 11.11 | 43.89 | 10.45 | 0.146 |
| Fine Motor  T-scores | 10 | 48.09 | 10.03 | 45.40 | 9.15 | 0.331 |
|  | 14 | 47.30 | 10.32 | 47.07 | 12.64 | 0.936 |
|  | 18 | 49.65 | 10.11 | 47.70 | 8.84 | 0.484 |
|  | 24 | 47.86 | 10.23 | 46.39 | 8.50 | 0.614 |
|  | 32 | 48.74 | 10.54 | 43.25 | 12.84 | 0.086 |
|  | 40 | 49.72 | 11.04 | 45.57 | 13.64 | 0.197 |
| Visual Reception  T-scores | 10 | 48.02 | 10.73 | 44.83 | 13.85 | 0.294 |
|  | 14 | 48.66 | 9.17 | 49.26 | 9.60 | 0.813 |
|  | 18 | 48.19 | 9.23 | 45.84 | 14.34 | 0.381 |
|  | 24 | 48.91 | 10.19 | 48.77 | 12.96 | 0.963 |
|  | 32 | 48.04 | 9.70 | 48.33 | 13.44 | 0.922 |
|  | 40 | 49.55 | 10.67 | 49.52 | 12.87 | 0.992 |
| Expressive Language  T-scores | 10 | 47.56 | 9.15 | 53.32 | 9.49 | 0.025 |
|  | 14 | 48.86 | 10.25 | 49.01 | 11.76 | 0.958 |
|  | 18 | 48.45 | 10.08 | 46.95 | 13.37 | 0.601 |
|  | 24 | 48.62 | 11.17 | 48.85 | 8.64 | 0.942 |
|  | 32 | 48.40 | 9.99 | 49.25 | 11.79 | 0.777 |
|  | 40 | 48.97 | 10.83 | 47.76 | 13.15 | 0.701 |
| Receptive Language  T-scores | 10 | 47.50 | 9.81 | 49.89 | 7.01 | 0.372 |
|  | 14 | 48.45 | 9.31 | 49.61 | 8.48 | 0.650 |
|  | 18 | 48.10 | 9.57 | 45.01 | 11.85 | 0.253 |
|  | 24 | 49.13 | 10.89 | 49.99 | 7.23 | 0.782 |
|  | 32 | 49.63 | 9.08 | 46.27 | 13.72 | 0.232 |
|  | 40 | 48.69 | 10.67 | 49.03 | 15.61 | 0.913 |

**I**

| Placental pathological finding of ‘Deciduitis’ | | | | | | |
| --- | --- | --- | --- | --- | --- | --- |
| month | | Deciduitis | | Deciduitis | | P |
|  |  | negative | | positive | |  |
|  |  | mean | Std. Dev | mean | Std. Dev |  |
| MSEL composite score | 10 | 97.94 | 14.97 | 95.32 | 17.27 | 0.546 |
|  | 14 | 97.56 | 13.46 | 96.04 | 14.49 | 0.707 |
|  | 18 | 99.74 | 15.88 | 97.42 | 10.69 | 0.592 |
|  | 24 | 98.50 | 12.15 | 97.56 | 9.02 | 0.786 |
|  | 32 | 98.50 | 14.39 | 100.01 | 9.88 | 0.702 |
|  | 40 | 97.76 | 15.07 | 104.63 | 14.43 | 0.112 |
| Gross Motor  T-scores | 10 | 45.71 | 9.71 | 42.95 | 10.77 | 0.308 |
|  | 14 | 47.74 | 11.34 | 48.02 | 11.81 | 0.931 |
|  | 18 | 48.18 | 10.06 | 48.46 | 9.06 | 0.918 |
|  | 24 | 47.49 | 9.359 | 47.71 | 7.04 | 0.932 |
|  | 32 | 47.89 | 10.33 | 48.86 | 14.00 | 0.743 |
|  | 40 | 48.07 | 10.99 | 50.46 | 12.79 | 0.439 |
| Fine Motor  T-scores | 10 | 47.97 | 9.97 | 47.03 | 10.45 | 0.744 |
|  | 14 | 47.32 | 10.27 | 46.75 | 13.36 | 0.849 |
|  | 18 | 49.48 | 10.07 | 50.01 | 9.70 | 0.850 |
|  | 24 | 47.79 | 10.16 | 47.41 | 9.84 | 0.892 |
|  | 32 | 48.25 | 10.95 | 50.46 | 7.52 | 0.459 |
|  | 40 | 49.22 | 11.25 | 52.85 | 10.79 | 0.244 |
| Visual Reception  T-scores | 10 | 48.10 | 10.94 | 43.79 | 10.79 | 0.156 |
|  | 14 | 48.77 | 9.27 | 47.61 | 7.91 | 0.673 |
|  | 18 | 48.03 | 9.64 | 48.04 | 9.93 | 0.995 |
|  | 24 | 48.83 | 10.38 | 49.94 | 10.14 | 0.699 |
|  | 32 | 48.10 | 10.06 | 47.54 | 8.13 | 0.839 |
|  | 40 | 49.27 | 10.92 | 53.61 | 7.90 | 0.161 |
| Expressive Language  T-scores | 10 | 47.87 | 9.17 | 49.20 | 10.94 | 0.618 |
|  | 14 | 49.03 | 10.16 | 46.46 | 12.89 | 0.406 |
|  | 18 | 48.41 | 10.54 | 47.46 | 6.57 | 0.739 |
|  | 24 | 48.64 | 11.18 | 48.52 | 8.34 | 0.971 |
|  | 32 | 48.29 | 10.24 | 50.50 | 7.59 | 0.432 |
|  | 40 | 48.67 | 10.98 | 51.90 | 10.60 | 0.289 |
| Receptive Language  T-scores | 10 | 47.89 | 9.56 | 44.29 | 10.85 | 0.194 |
|  | 14 | 48.54 | 9.32 | 48.51 | 8.13 | 0.992 |
|  | 18 | 48.03 | 9.83 | 45.98 | 8.57 | 0.450 |
|  | 24 | 49.35 | 10.58 | 47.09 | 12.20 | 0.446 |
|  | 32 | 49.34 | 9.55 | 50.50 | 7.60 | 0.658 |
|  | 40 | 48.38 | 11.00 | 53.30 | 10.21 | 0.106 |

**J**

| Placental pathological finding of ‘Maternal vascular malperfusion’ | | | | | | |
| --- | --- | --- | --- | --- | --- | --- |
| month | | Maternal vascular malperfusion | | Maternal vascular malperfusion | | P |
|  |  | negative | | positive | |  |
|  |  | mean | Std. Dev | mean | Std. Dev |  |
| MSEL composite score | 10 | 99.98 | 14.40 | 95.36 | 15.52 | 0.028 |
|  | 14 | 98.00 | 11.77 | 96.91 | 15.11 | 0.582 |
|  | 18 | 101.48 | 15.36 | 97.55 | 15.58 | 0.077 |
|  | 24 | 99.36 | 11.57 | 97.34 | 12.35 | 0.241 |
|  | 32 | 100.01 | 13.28 | 97.11 | 14.86 | 0.152 |
|  | 40 | 100.30 | 14.42 | 95.96 | 15.53 | 0.040 |
| Gross Motor  T-scores | 10 | 46.85 | 9.13 | 44.04 | 10.30 | 0.039 |
|  | 14 | 48.52 | 10.88 | 46.94 | 11.82 | 0.327 |
|  | 18 | 47.79 | 9.12 | 48.64 | 10.84 | 0.543 |
|  | 24 | 48.41 | 8.857 | 46.49 | 9.53 | 0.139 |
|  | 32 | 48.04 | 10.49 | 47.88 | 10.77 | 0.918 |
|  | 40 | 49.15 | 10.76 | 47.28 | 11.42 | 0.226 |
| Fine Motor  T-scores | 10 | 48.91 | 9.63 | 46.80 | 10.28 | 0.129 |
|  | 14 | 47.22 | 9.67 | 47.36 | 11.30 | 0.927 |
|  | 18 | 49.65 | 10.58 | 49.36 | 9.427 | 0.839 |
|  | 24 | 48.62 | 10.29 | 46.82 | 9.89 | 0.204 |
|  | 32 | 49.55 | 10.86 | 47.15 | 10.52 | 0.118 |
|  | 40 | 51.47 | 11.14 | 47.42 | 11.01 | 0.009 |
| Visual Reception  T-scores | 10 | 48.67 | 10.48 | 46.85 | 11.47 | 0.235 |
|  | 14 | 48.27 | 9.64 | 49.21 | 8.67 | 0.459 |
|  | 18 | 48.62 | 9.59 | 47.38 | 9.68 | 0.361 |
|  | 24 | 49.86 | 10.41 | 47.88 | 10.22 | 0.168 |
|  | 32 | 48.62 | 9.42 | 47.44 | 10.46 | 0.404 |
|  | 40 | 50.51 | 10.21 | 48.53 | 11.32 | 0.189 |
| Expressive Language  T-scores | 10 | 49.00 | 9.66 | 46.91 | 8.72 | 0.092 |
|  | 14 | 49.86 | 9.47 | 47.80 | 11.12 | 0.166 |
|  | 18 | 48.78 | 9.77 | 47.89 | 10.88 | 0.544 |
|  | 24 | 49.53 | 10.54 | 47.62 | 11.49 | 0.222 |
|  | 32 | 49.81 | 9.07 | 46.99 | 10.91 | 0.049 |
|  | 40 | 49.54 | 10.61 | 48.23 | 11.32 | 0.390 |
| Receptive Language  T-scores | 10 | 48.76 | 9.358 | 46.44 | 9.87 | 0.085 |
|  | 14 | 48.80 | 8.89 | 48.27 | 9.60 | 0.695 |
|  | 18 | 49.33 | 9.52 | 46.36 | 9.79 | 0.031 |
|  | 24 | 50.24 | 10.07 | 47.98 | 11.28 | 0.139 |
|  | 32 | 49.91 | 8.32 | 48.90 | 10.50 | 0.457 |
|  | 40 | 49.81 | 10.75 | 47.58 | 11.17 | 0.142 |

**K**

| Placental pathological finding of ‘Fetal vascular malperfusion’ | | | | | | |
| --- | --- | --- | --- | --- | --- | --- |
| month | | Fetal vascular malperfusion | | Fetal vascular malperfusion | | P |
|  |  | negative | | positive | |  |
|  |  | mean | Std. Dev | mean | Std. Dev |  |
| MSEL composite score | 10 | 95.74 | 15.38 | 101.62 | 13.83 | 0.008 |
|  | 14 | 96.08 | 14.23 | 100.04 | 11.69 | 0.056 |
|  | 18 | 97.49 | 15.37 | 103.59 | 15.22 | 0.009 |
|  | 24 | 97.12 | 11.24 | 100.76 | 12.85 | 0.041 |
|  | 32 | 97.44 | 13.77 | 100.80 | 14.56 | 0.113 |
|  | 40 | 96.71 | 14.07 | 101.15 | 16.66 | 0.047 |
| Gross Motor  T-scores | 10 | 45.08 | 9.59 | 46.36 | 10.15 | 0.371 |
|  | 14 | 47.11 | 11.51 | 48.96 | 11.00 | 0.275 |
|  | 18 | 47.79 | 9.95 | 49.00 | 10.05 | 0.412 |
|  | 24 | 47.39 | 9.22 | 47.72 | 9.23 | 0.802 |
|  | 32 | 48.22 | 10.16 | 47.49 | 11.42 | 0.649 |
|  | 40 | 47.65 | 10.92 | 49.30 | 11.43 | 0.308 |
| Fine Motor  T-scores | 10 | 46.73 | 10.06 | 50.10 | 9.50 | 0.021 |
|  | 14 | 47.06 | 10.73 | 47.72 | 10.00 | 0.672 |
|  | 18 | 48.56 | 9.619 | 51.37 | 10.59 | 0.059 |
|  | 24 | 46.78 | 9.88 | 49.53 | 10.36 | 0.061 |
|  | 32 | 47.79 | 10.05 | 49.54 | 11.90 | 0.278 |
|  | 40 | 48.63 | 10.64 | 51.01 | 12.20 | 0.150 |
| Visual Reception  T-scores | 10 | 46.70 | 11.31 | 49.89 | 10.02 | 0.046 |
|  | 14 | 48.06 | 9.04 | 49.93 | 9.39 | 0.173 |
|  | 18 | 47.21 | 9.61 | 51.97 | 10.97 | 0.001 |
|  | 24 | 47.04 | 8.85 | 49.96 | 10.80 | 0.040 |
|  | 32 | 47.21 | 9.61 | 51.97 | 10.97 | 0.001 |
|  | 40 | 46.97 | 9.79 | 50.08 | 9.91 | 0.036 |
| Expressive Language  T-scores | 10 | 46.70 | 9.12 | 50.33 | 9.15 | 0.007 |
|  | 14 | 47.60 | 11.21 | 51.27 | 7.95 | 0.018 |
|  | 18 | 47.33 | 10.49 | 50.36 | 9.71 | 0.048 |
|  | 24 | 48.13 | 10.54 | 49.56 | 11.84 | 0.381 |
|  | 32 | 47.94 | 10.13 | 49.42 | 9.97 | 0.327 |
|  | 40 | 47.80 | 10.42 | 50.95 | 11.70 | 0.048 |
| Receptive Language  T-scores | 10 | 47.23 | 9.97 | 48.45 | 9.05 | 0.389 |
|  | 14 | 47.88 | 9.49 | 49.74 | 8.68 | 0.186 |
|  | 18 | 46.82 | 9.45 | 49.93 | 10.04 | 0.032 |
|  | 24 | 48.68 | 10.67 | 50.08 | 10.72 | 0.378 |
|  | 32 | 49.03 | 8.80 | 50.17 | 10.51 | 0.424 |
|  | 40 | 47.82 | 10.24 | 50.37 | 12.17 | 0.110 |

**Supplemental table2**

**Correlation with MSEL composite scores**

| Month of age | 10 | 14 | 18 | 24 | 32 | 40 |
| --- | --- | --- | --- | --- | --- | --- |
| **Parent characteristics** |  |  |  |  |  |  |
| Maternal age (yr.)  (P value) | 0.071  (0.32) | 0.014  (0.84) | 0.105  (0.14) | 0.036  (0.62) | 0.005  (0.95) | 0.040  (0.59) |
| Maternal body weight (non-pregnant) (kg)  (P value) | -0.065  (0.35) | -0.060  (0.41) | -0.084  (0.24) | -0.121  (0.09) | -0.048  (0.50) | -0.136  (0.07) |
| Maternal BMI (non-pregnant) (kg/m^2^)  (P value) | -0.084  (0.23) | -0.085  (0.24) | -0.106  (0.14) | -0.16  (0.03) | -0.099  (0.17) | -0.206  (0.01) |
| Body weight gain during pregnancy (kg)  (P value) | 0.063  (0.37) | 0.074  (0.31) | 0.001  (0.99) | 0.052  (0.48) | 0.034  (0.64) | 0.032  (0.67) |
| Household income (million JPY/year)  (P value) | 0.025  (0.73) | -0.055  (0.45) | 0.042  (0.56) | 0.094  (0.19) | 0.121  (0.09) | 0.131  (0.08) |
| Maternal education (Year)  (P value) | 0.081  (0.25) | -0.012  (0.87) | 0.080  (0.27) | 0.100  (0.17) | 0.126  (0.08) | 0.142  (0.06) |
| Paternal age (yr.)  (P value) | -0.099  (0.16) | -0.098  (0.17) | -0.03  (0.68) | -0.02  (0.81) | -0.037  (0.61) | -0.051  (0.50) |
| Hypertension during pregnancy  (P value) | -0.110  (0.12) | -0.138  (0.06) | -0.099  (0.17) | -0.058  (0.42) | -0.063  (0.38) | -0.192  (0.01) |
| Postpartum depression (3 months after child birth)  (P value) | -0.037  (0.60) | -0.081  (0.26) | -0.020  (0.78) | -0.107  (0.14) | -0.057  (0.43) | -0.014  (0.85) |
| **Infant characteristics** |  |  |  |  |  |  |
| Birth weight (g)  (P value) | 0.110  (0.12) | 0.131  (0.07) | 0.236  (0.001) | 0.253  (0.00) | 0.189  (0.01) | 0.245  (0.001) |
| Gestational age at birth  (P value) | 0.220  (0.002) | 0.124  (0.09) | 0.221  (0.002) | 0.235  (0.001) | 0.137  (0.06) | 0.159  (0.03) |
| Umbilical arterial pH  (P value) | -0.197  (0.006) | -0.179  (0.02) | -0.127  (0.09) | -0.043  (0.56) | -0.046  (0.53) | 0.017  (0.83) |

The upper row is correlation coefficient.

Supplemental Table3

**A Individual pathological observations of the placenta**

|  | **Model 2** | **Model 4a** | **Model 4b** | **Model 4c** |
| --- | --- | --- | --- | --- |
|  | **Coefficient (95% Conf. Interval)** | **Coefficient (95% Conf. Interval)** | **Coefficient (95% Conf. Interval)** | **Coefficient (95% Conf. Interval)** |
| Accelerated villous maturation^#^ | -2.57  (-4.45 to -0.69) | -2.55  (-4.43 to -0.68) | -2.30  (-4.18 to -0.42) | -2.52  (-4.42 to -0.63) |
| Decidual arteriopathy | -0.37  ( -2.07 to 1.32) | -0.30  (-2.00 to 1.39) | -0.54  (-2.24 to 1.15) | -0.37  (-2.07 to 1.32) |
| Thrombosis or intramural fibrin deposition^$^ | 2.51  (0.72 to 4.31) | 2.71  (0.92 to 4.50) | 2.45  (0.66 to 4.24) | 2.52  (0.72 to 4.32) |
| Avascular villi^$^ | 2.50  (-0.13 to 5.13) | 1.9  (-0.73 to 4.56) | 2.47  (-0.14 to 5.09) | 2.52  (-0.11 to 5.14) |
| Delayed villous maturation^#^ | -2.87  (-4.83 to -0.91) | -3.00  (-4.96 to -1.05) | -2.98  (-4.93 to -1.02) | -2.84  (-4.80 to -0.87) |
| Maternal inflammatory response | 0.12  (-1.74 to 1.98) | 0.37  (-149 to 2.23) | 0.42  (-1.44 to 2.29) | 0.13  (-1.73 to 1.99) |
| Fetal inflammatory response^$^ | 1.02  (-1.31 to 3.36) | 1.00  (-1.32 to 3.32) | 0.57  (-1.77 to 2.91) | 0.96  (-1.39 to 3.31) |
| Villitis of unknown etiology | -2.24  (-5.41 to 0.93) | -2.48  (-5.65 to 0.68) | -2.78  (-5.95 to 0.40) | -2.29  (-5.47 to 0.89) |
| Deciduitis | -0.89  (-4.03 to 2.24) | -0.69  (-3.82 to 2.43) | -1.01  (-4.13 to 2.12) | -0.92  (-4.06 to 2.22) |

**B Conceptional pathological diagnosis of the placenta**

|  | **Model 3** | **Model 4a** | **Model 4b** | **Model 4c** |
| --- | --- | --- | --- | --- |
|  | **Coefficient (95% Conf. Interval)** | **Coefficient (95% Conf. Interval)** | **Coefficient (95% Conf. Interval)** | **Coefficient (95% Conf. Interval)** |
| Maternal vascular malperfusion^#^ | -2.12  (-3.71 to -0.54) | -2.10  (-3.67 to -0.52) | -2.01  (-3.58 to -4.28) | -2.06  (-3.66 to -0.46) |
| Fetal vascular malperfusion^$^ | 3.43  (1.77 to 5.09) | 3.48  (1.83 to 5.13) | 3.26  (1.60 to 4.91) | 3.42  (1.76 to 5.07) |

Significance was set at a p value of 0.05 as described in the Methods. ^#^: significant predictors of the relatively delayed achievement of neurodevelopmental milestones. ^$^: significant predictors of the relatively faster achievement of neurodevelopmental milestones.

The regression coefficients represent any changes associated with the presence/absence of the specific placental pathological changes, measured with the Mullen Scales of Early Learning composite score with the mean of 100 and the standard deviation of 15.

Model 1; Covariates included in the analysis were maternal parity, birth weight, and infantile sex, which may be related to placental pathological findings and MSEL composite scores.

Model 2; Additional covariates of all of other placental pathological findings.

Model 3; Additional covariate of all of Fetal vascular malperfusion or Maternal vascular malperfusion.

Model 4a; Additional covariate of Maternal education.

Model 4b; Additional covariate of Hypertension during pregnancy.

Model 4c; Additional covariate of Gestation diabetes mellitus.
